# Ductal Architecture: Corrosion Casting of Canine Mammary Glands Using an Intraductal Approach

**DOI:** 10.3390/ani13182932

**Published:** 2023-09-15

**Authors:** Sabina Sibcic Kolasinac, David Griffiths, Lars Moe, Henning Sørum, Vibeke Rootwelt

**Affiliations:** 1 Department of Companion Animal Clinical Sciences, Faculty of Veterinary Medicine, Norwegian University of Life Sciences, Elizabeth Stephansens vei 15, 1433 Aas, Norway; lars.moe@nmbu.no (L.M.); vibeke.rootwelt@nmbu.no (V.R.); 2 Department of Companion Animal Clinical Sciences, Veterinary Faculty, University of Sarajevo, Zmaja od Bosne 90, 71000 Sarajevo, Bosnia and Herzegovina; 3 Department of Preclinical Sciences and Pathology, Faculty of Veterinary Medicine, Norwegian University of Life Sciences, Elizabeth Stephansens vei 15, 1433 Aas, Norway; david.griffiths@nmbu.no; 4 Department of Paraclinical Sciences, Faculty of Veterinary Medicine, Norwegian University of Life Sciences, Elizabeth Stephansens vei 15, 1433 Aas, Norway; henning.sorum@nmbu.no

**Keywords:** descriptive canine anatomy, 3D milk ducts, resin perfusion, mammary gland replica

## Abstract

**Simple Summary:**

Milk is a natural and essential nutrient for new-born mammals. Dogs were one of the first domesticated species to coexist with humans, and yet scientists lack some crucial information related to the anatomy and morphology of the canine mammary gland, the organ after which all mammals are named. This study investigated mammary glands’ inner structures using intraductal corrosion casting. We intended to provide visualisation of the glandular and ductal structures and their branching presented as a three-dimensional image. The aim was to expand current knowledge in the anatomy field by describing the in situ position (i.e., the undisturbed shape and natural position) of glandular tissue and its ductal branching pattern. No obvious teat cistern was present. Glandular branch intertwining of different gland lobes without intercommunication was observed. Ascendant teat canals could drain single or multiple gland lobes or could end blind. Although it had a small sample size, this study confirmed that the intraductal cannulation of healthy canine mammary glands is possible through functional and developed orifices. New knowledge in this field could contribute to the better management and understanding of neoplastic and non-neoplastic conditions that veterinary clinicians and surgeons often encounter.

**Abstract:**

Detailed knowledge related to the morphology, anatomy, physiology, and pathology of the canine mammary gland is scarce. Mammary tissue undergoes massive changes instructed by hormones multiple times within the lifespan of every bitch, affecting its appearance. To address the ductal system’s appearance and to present how different our findings regarding the canine mammary gland are in comparison with the available literature, we obtained cadaveric specimens after euthanasia and mastectomy. All bitches were euthanised due to poor prognosis for their recovery from maladies unrelated to mammae. Using intraductal cannulation ex vivo, milk- or fluid-yielding ducts were perfused using VasQtec (polyurethane resin), which revealed casts, i.e., imprints of ducts and glandular structures in situ. We observed primary, vertically positioned ducts that ascended throughout the teat and continued branching to secondary, tertiary, etc., horizontally positioned ducts, which drained mammary gland lobes under the skin located close to the abdominal wall. The ascendant teat canal could be split into two and could be connected to gland alveoli or end blind. Alveolar formations were located along ducts and ductules in bigger and/or smaller clusters. This study is the first to generate a 3D image of canine ducts and glandular tissue using an intraductal approach.

## 1. Introduction

Milk is essential for offsprings’ survival, but scientists still lack crucial information about canine mammary glands (milk-producing organs). Compared with the present knowledge related to the morphology, anatomy, physiology and pathology of the mammary glands in ruminants and humans, similar scientific data for carnivores are scarce [1]. In the available literature, the canine mammary gland complex is described as a set of modified exocrine skin glands, located bilaterally on the ventral side, stretching from the axillar to the inguinal region [2,3]. In the early embryonic phase, a developmental stage has been described in which two longitudinal ectodermal mammary lines develop into placodes. Interactions between epithelial and mesenchymal tissue lead to an embedding of the placodes into the underlying mesenchymal tissue where ductal structures and glands develop [4]. This process is independent of hormones. Once developed, mammary glands remain dormant until the onset of puberty; when instructed by hormones, the process of massive changes in morphology and functionality begins. The proliferation and regression of glandular tissues and their ductal and vascular systems alternate during pregnancy, lactation, involution and within each oestrous cycle [5,6,7].

There are usually five pairs of glands; the term “gland” is defined as the secretory units and associated ducts and orifices on a single teat (two thoracic (T1 and T2), two abdominal (A1 and A2) and one inguinal pair (I)) [8], although deviation from this number also occurs. Each gland consists of a few compartments, with its own glandular lobe(s) and orifices on the teat, and acts as an independent functional unit withing the gland. The teat receives 8–22 duct openings around the teat apex periphery [9], each of which drains a lobe of secretory tissue within the gland [10]. The canine mammary gland’s three-dimensional anatomical structure is poorly described in the literature. In 1939, Turner described how the teat openings led into “streak canals”, which developed into “teat cisterns” that continued down the whole length of the teat into the gland parenchyma [9]. One dissertation [11] described streak canals or cisterns that might split into two or several sections on their way towards the gland, but that all of these ended blind and did not connect to gland alveoli. A century later, the current scientific literature still does not provide detailed insight into the ductal system’s architecture, and the existing schematic drawings [12] are not based upon detailed three-dimensional studies. Whether a single duct can drain multiple gland lobes is also uncertain.

The first known cast of the internal cavity of a mammal organ was made by Leonardo da Vinci in the 16th century when he injected beeswax into bovine brain ventricles to make an imprint. The method has been improved several times over the years and, nowadays, corrosion casts are resistant polymer residuals that remain after a cavity is filled with a material that subsequently hardens, after which the surrounding tissue is macerated in caustic solutions.

This descriptive study aimed to determine (a) whether it was possible to visualise the finer ductal structures of a dog’s mammary gland using the standard corrosion cast technology; (b) the presence and forms of the teat sinus and gland cistern; (c) whether ducts from separate teat orifices in the same teat communicate; (d) whether every milk duct always drains a single, associated excretory gland compartment; and (e) to compare two different casting techniques.

## 2. Materials and Methods

### 2.1. Tissue for Study

Strips of ventral abdominal and caudal thoracic body wall, including skin, subcutaneous tissue and glands, were collected after euthanasia due to poor prognosis related to diseases and conditions unrelated to mammary gland involvement, such as polyneuropathy, chronic renal disease, cancer, heart failure and financial constraints. Five bitches aged 8 to 13 years (mean = 11 years), of different breeds (Alaskan Malamute, Flat-Coated Retriever, Cavalier King Charles Spaniel, German Shorthaired Pointer and mixed breed) and in different oestrus phases were included in this study. One was in pseudocyesis. Mastectomy samples included five complete blocks of tissue, four of which had five pairs of glands and one with four pairs of glands, i.e., a total of 48 single mammary glands. One complete set of glands from one bitch (10/48) was assessed intact to check the in situ position of ducts in different glands on both sides. The remaining samples (38/48) were cut and assessed as either a single gland or as a set of 2, 3, 4 or 5 neighbouring glands depending on the method we followed. Written consent was obtained from all dog owners. Background information, such as previous litters or oestrus cycle, was desirable, but not required inclusion criteria. Tissue blocks were subsequently stored in a freezer at −20 °C until perfusion. Hair was removed with clippers before mastectomies were performed.

### 2.2. Ex Vivo Casting of the Mammary Gland

Each block of the ventral body wall was thawed at room temperature for 2 h prior to casting. The apex of each teat was inspected under a Leica MZ6 stereo dissection microscope(Leica Microsystems, Heerbrugg, Switzerland) to locate duct orifices and clean them of sebum and keratinised epidermal residue using warm water and 70% ethanol. To help locate duct orifices, each teat was gently compressed between the thumb and forefinger to force liquid that might be in the ducts up to the skin surface.

**Figure 1:**
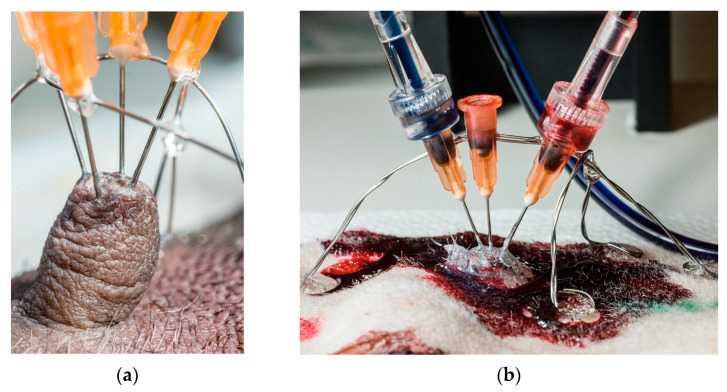
The preparation process for resin injection. (**a**) Placement of stainless stirrups prior to needles’ fixation. The entire construction was additionally fixed with glue, making it stable enough for syringe mounting and perfusion. (**b**) Resin injection technique.

Needles (0.515 mm (25 g) × 1.5 cm) were used to introduce resin into the teat canals. Each needle’s sharp tip was removed and rounded using a grinding wheel; the needle’s shaft was roughened to provide a better grip for the glue used. Needles’ placement in teats is shown in Figure 1.

Between one and four ducts were perfused per teat, using one needle per duct opening. Thick cyanoacrylate glue (Biltema, Oslo, Norway) was applied around the base of each teat and partly up its sides to support and stiffen the teat. Before needles were placed, two stainless steel semi-circular hoops of stainless steel were formed and placed over each teat, at right angles to each other, and glued to the skin with cyanoacrylate glue. As the needle’s diameter was greater than that of the teat ducts’ orifices, the needle’s tip was placed as snugly as possible against the teat opening, and the needle’s shaft was glued onto the supporting steel wire hoops. When all needles were in place, the entire teat, the bases of all needles and the surrounding skin were covered with thick cyanoacrylate glue to lock and seal the needles against the skin, and to create a reference surface to show where the skin had been, after corrosion.

We used two types of casting material for perfusion. PU4ii, a polyurethane resin developed specifically for corrosion casting (VasQtec, Zürich, Switzerland), was used to produce high-quality casts of the teat and gland ducts for scanning electron microscopy (SEM). PU4ii has a working time of 10–30 min. Epoxy casting and laminating resin has a several-hour working time in wet tissue; therefore, we used it to fill as much of the duct network as possible, so as to provide the best macroscopic overview of the size and nature of the duct network feeding to a single teat duct.

### 2.3. PU4ii

In total, 5 g of infusion resin was mixed with 3 g of butanone (to reduce its viscosity) in a glass vial with a neoprene cap. Blue or red pigment was added (0.03 g) and mixed thoroughly until homogenised. VasQtec fluorescent dye (10–20 mg) was added to improve fluorescence. Immediately before perfusion, VasQtec hardener (0.08 g) was added and mixed.

### 2.4. Epoxy Resin

A total of 10 g of epoxy resin (RSF 816) was mixed with 0.7 g of butanone in a glass vial with a neoprene cap. White or yellow dye (0.1 g) was added and dissolved in the liquid using an electric mixer. Using different-coloured pastes simplified the visualisation and differentiation of ducts when more than one duct was perfused on a teat. Just before perfusion, 4 g of hardener (RSF816) was added.

Perfusion was manual, using a 1 mL or 2 mL Luer disposable syringe. A three-way stopcock with a 15 cm extension tube was fitted between the syringe and the needle. The resin was forced from the syringe into the extension until it was full, and then the resin was carefully dripped into the needle socket until it was also full. Next, the extension was tightly fitted onto the needle socket and perfusion was initiated at approximately 0.1 mL/min. PU4ii infusion was stopped when the resin began to thicken. Epoxy resin infusion was stopped when significant resistance was felt; the extension was then pressurised by forcing more epoxy into it until visible distension was seen. It was hoped that epoxy would continue to move through the duct system for some time, for maximal filling. A new syringe and extension were used for each duct infusion.

Tissue blocks were left undisturbed for up to 24 h, until hardening was complete. They were then immersed skin-side down in a bath of 10% potassium hydroxide (KOH) at room temperature for 24–48 h, and then carefully rinsed with tap water until all tissue was removed. Tissue blocks were etched with the skin down so that the cyanoacrylate glue that had been spread over the skin surface and hardened would then support the casts. After all the tissue had dissolved and air-drying was complete, successful corrosion casts were mounted on aluminium stubs and assessed with a magnifying glass using light and electron microscopy.

## 3. Results

**Figure 2:**
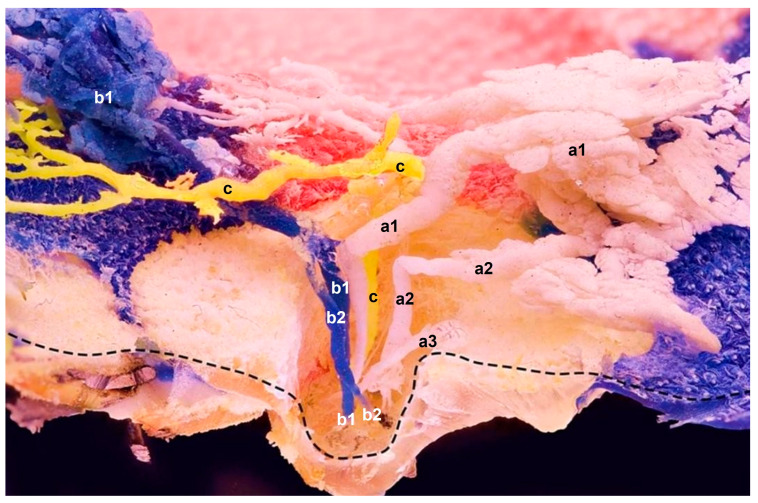
Sagittal plane of the epoxied mammary gland with three resin applications (**a**–**c**) using three needles in three separate orifices at a time. The main ducts’ sharp curvatures show no noticeable duct expansions. Instead, ascendant ducts of three different glandular compartments show (**c**) single and (**a**,**b**) multiple branches within the teat; their spreading and branching expose their in situ anatomical positions. **a**—One orifice and multiple ducts, which drain associated gland lobes within one compartment (**a1** and **a2** are functional ducts with their own glandular sections); **a3** has no glandular section attached and ends blind; **b**—Multiple ducts (**b1** and **b2**) that coalesce at the curvature location. Resin probably travelled upwards via **b1**, and then transferred to **b2**. One injection site, but two orifices; **c**—One orifice and a single duct that drains a single compartment. Mere duct with no attached alveoli; **------** indicates the preserved superficial skin layer.

Using an intraductal approach through the teat orifices, we produced casts showing in situ positions of milk ducts and alveoli within the glandular tissue. Instead of a cistern, we observed two types of ducts: (a) ascending ducts that gradually expended and were three times the size of the teat orifice at its widest diameter; and (b) small ducts with uniform widths through the entire teat. After ascending vertically for approximately 1 cm, ducts turned at 90-degree angles in the cranial or caudal direction, depending on which orifice was cannulated. Ducts spread out superficially under the skin, flat and close to the abdominal wall. The ascendant teat canal could split into two or three sections, and could be connected to the glandular tissue with alveoli or end blind, as presented in Figure 2. Ducts’ diameters were widest just before they connected to the glandular tissue.

**Figure 3:**
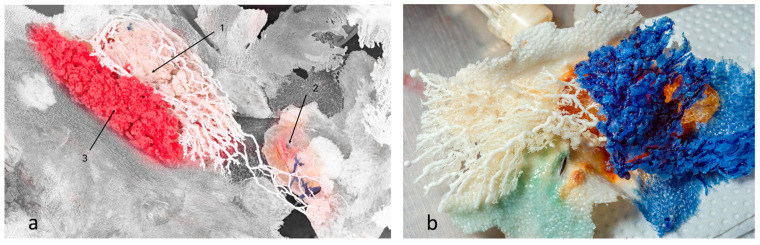
Mammary glands with preserved skin surface, positioned in situ. (**a**) Resins of different glands interlaced, but did not communicate or mix. Branches of white resin stretched to another gland’s location. Section 1 and Section 3 are the first and second compartments of one gland, respectively, whereas Section 2 is the first (underdeveloped) compartment of another gland (blue resin). The grey area represents preserved skin surface. (**b**) Two compartments of the same gland. No resin mixing was reported. Preserved skin texture was also noted.

However, we observed that the ductal branches of one gland could connect with a neighbouring gland, causing interlacing of ducts and glandular tissue, i.e., compartments of different glands could be found at the same location with no observed mixing of resin between duct systems (Figure 3a). On the other hand, we also observed examples of no interlacing of ducts or glandular tissue among an individual gland’s compartments (Figure 3b).

**Figure 4:**
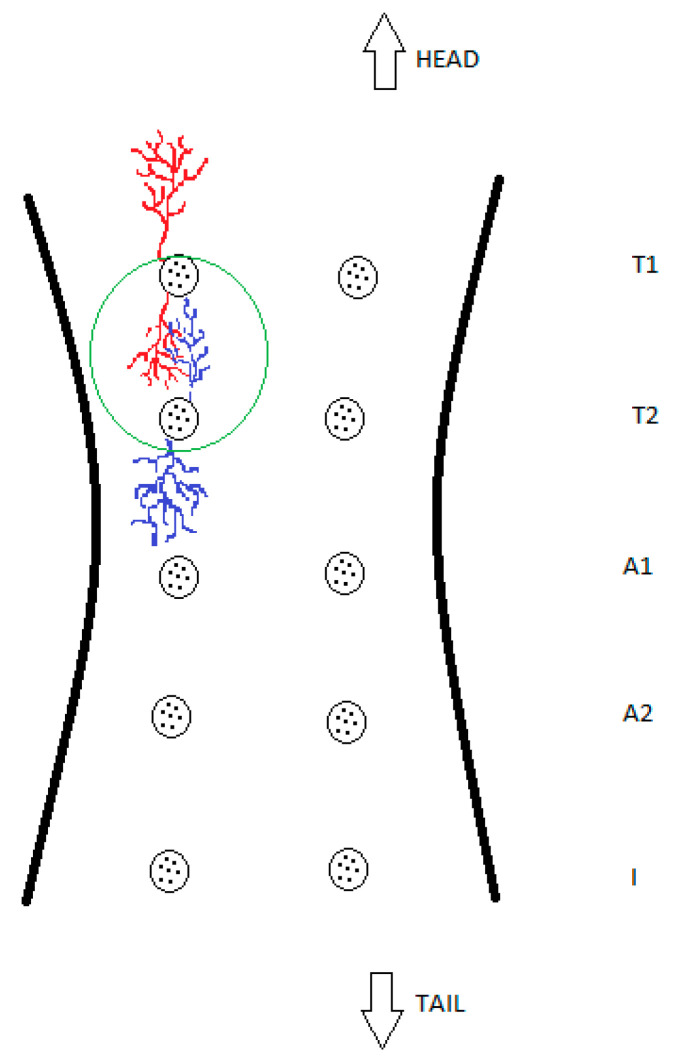
Schematic drawing of glandular overlapping, which demonstrates and simplifies this study’s findings. The green circle shows the intertwining of two glandular compartments of different glands with no observed mixing of blue and red coloured resin or their intercommunication.

For further clarification, these findings are also presented schematically in Figure 4.

**Figure 5:**
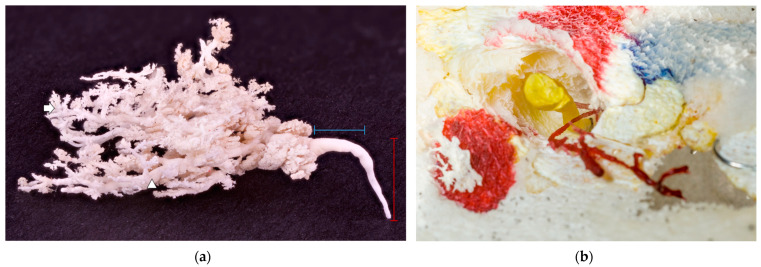
(**a**) A white glandular cast on a black background. The red label represents an ascendant milk duct within a teat, and the blue label represents a transversal, wider milk duct just before it started to branch. No obvious teat cistern was present; milk duct deeper in the glandular parenchyma (arrowhead); and alveoli (arrow). (**b**) A blind-ended duct filled with yellow resin and an underdeveloped duct with no glandular tissue attached filled with red resin. The teat’s hollow structure is easily seen around the yellow resin, preserved in the casting process for easier orientation.

A functional and developed ductal system was required for successful cast production. Representative in situ casts with obvious ducts and further branching were obtained by injecting multiple orifices using different-coloured resins. Injecting a maximum of two needles simultaneously provided the best results; more than two needles was often too many, as the glue leaked over to and interfered with neighbouring needles. One cast of mammary glands detached from the skin is shown in Figure 5a. The same resin injection procedure was performed on all orifices, but some still ended blind (Figure 5b).

**Figure 6:**
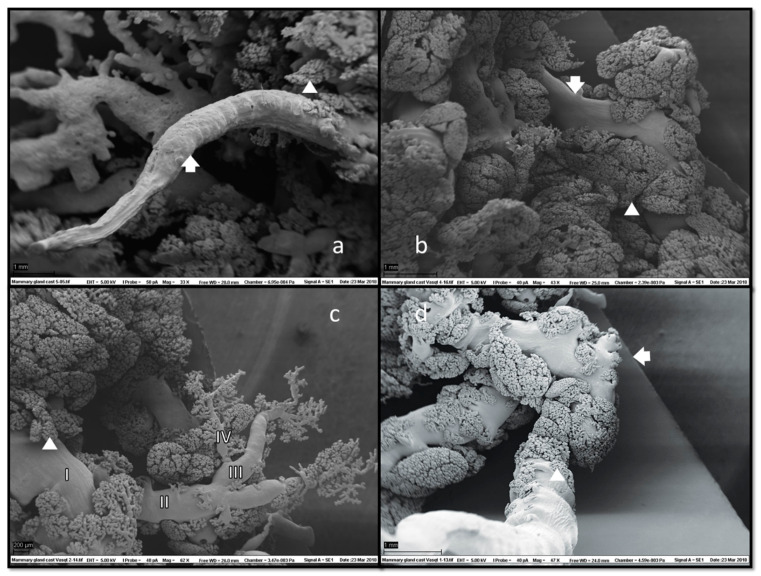
Scanning electron microscopy (SEM) of mammary gland casts revealed fine ductal structures (arrows) with adjacent glandular tissue (**a**,**b**). Alveoli (arrowheads) morphology was clearly visible, usually located marginally, i.e., on the tiniest branches of ducts and ductules and along their entire length (**c**). Duct branching started halfway down the teat duct’s ascending section (**d**), which could also have a collapsed appearance (**a**). No duct cisterns were visible. Magnifications: (**a**) 33×, (**b**) 43×, (**c**) 62× and (**d**) 47×.

In SEM of corrosion casts (Figure 6), grape-like formations of ducts and ductules with attached alveoli were located superficially under the skin.

**Figure 7:**
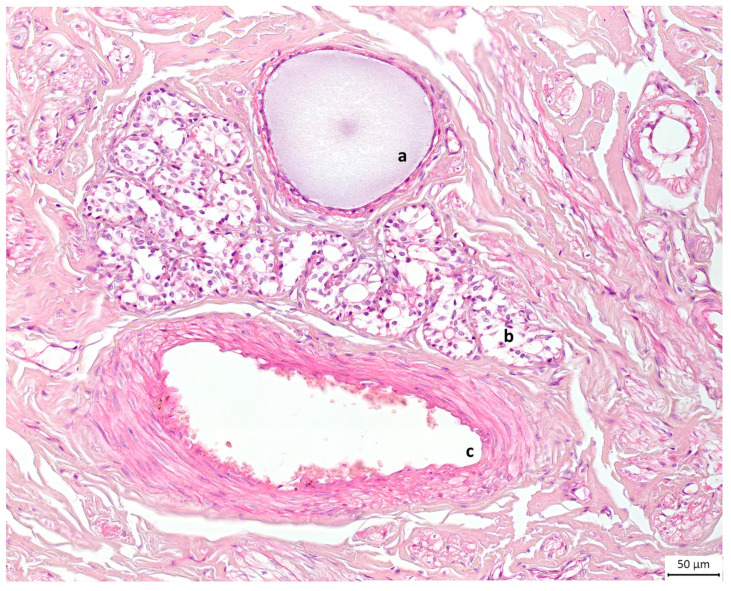
Light microscopic view of canine mammary tissue. This cross section shows (**a**) a duct filled with milk and (**b**) glandular tissue composed of alveoli next to (**c**) the blood supply; the rest is connective tissue or stroma. Haematoxylin and eosin stain. Original magnification ×20.

Alveolar formations of glandular tissue were located along ducts of different calibres. Figure 6d shows that glandular tissue started to form halfway down the duct, and then the duct made a 90-degree angle turn from vertical to horizontal. The same structural disposition is noted on a histology slide preparation presented in Figure 7.

## 4. Discussion

This study generated fine imprints of ducts and glandular structures in canine mammary glands using the intraductal approach of the corrosion casting technique. In modern science, casts have been used to clarify, describe and present 3D structures of ducts or vessels of various organs for decades [13,14,15,16,17,18]. Regarding our choice of casting material, we used two types, hoping for a better results comparison. As we expected based on the research [19], the polyurethane-based casting resin (PU4ii) provided casts with finer detail imprints. It had superior viscosity and shrinkage characteristics. A short working period due to its quick hardening was PU4ii’s only downside; however, projections of ductal and glandular details were impeccable if small sections of ducts were injected at a time.

In our study, using milk- or fluid-yielding orifices yielded more successful casts. With the onset of pregnancy, glandular tissue starts to grow. Ten days after parturition, it starts to regress and involutes after puerperium ends (12 weeks postpartum). Therefore, glandular growth is oestrogen–progesterone-dependent [20]. It was easier to produce casts in bigger glands. Attempts to make casts using cadaveric specimens of non-lactating glands (i.e., glands in the proestrus and late anoestrous stages) were not as successful, and provided only poor ductal branching images. Notably, when comparing our results to those of older studies [9,11], no obvious cistern was found in the ductal system.

Among produced casts, some were shorter or less developed. Why were some ducts and glands better developed? During the non-lactating phase, canine mammary glands are typically characterised by collapsed teat ducts and lower density vascularisation, resulting in smaller teats [21]. There were also individual variations in gland development; the number of alveoli per lobule in gland no. 3 (or abdominal gland no. 1; A1) was smaller than it was in the first two pairs of thoracic glands. The number of alveoli per se is proportional to milk production [22]. Lower milk production in gland no. 3 implied earlier involution of the gland, which resulted in a small, underdeveloped gland and non-representative casts. Another study of the mammary gland’s microscopic anatomy reported that the gland of an adult female dog is inactive during the proestrus stage, when it primarily consists of interlobular ducts. In late anoestrus, duct lumina are decreased in diameter, which is in agreement with previous findings [23]. This may explain why we obtained better casts from lactating specimens or specimens in the oestrus and dioestrus stages and why we found both types of ducts.

It might also be that the perfusion did not target a dominant duct, or hit blind-ended ducts. Those ducts were also previously described as rudimentary or atrophic ducts, i.e., structures that go through the nipple but do not enter the lobe and have no ductal function [24,25]. This is consistent with our finding presented in Figure 4. It could also be that, although we cleaned and manually extracted keratin plugs out of blocked ducts, some particles remained and were pushed deeper into the lumen; this could have eventually stopped the perfusion. The observation of blind-ended milk ducts fits with the reported embryological development, during which milk ducts develop via epithelial cells growing from the teat into the mesenchyme, where they are met by vascular endothelial cells and fibroblasts in the subcutaneous fat tissue [4]. Thus, blind-ended milk ducts could be described as a product of minor malformations due to disturbances in the neat process of ductal system development during the gestation period.

Regarding teat orifices and ascendant ducts, we observed 5 to 6 functional orifices, and occasionally a few more that were non-functional or filled with keratin plugs, in comparison with previous findings, which described up to 22 teat orifices [9]. Figure 2 illustrates that ascendant teat ducts could be solitary or split into two or three sections; of those three ducts, two had drained two glandular lobes, and one ended blind. In addition to the teat’s ascendant section, ducts could also split at the curvature location, where the glandular tissue lobation starts. What began as a single duct, split and ended at a different orifice once it reached the curvature location; thus, two ducts from two teat orifices drained the same gland lobe. This might be one reason the literature describes a higher number of orifices in comparison with the number of glandular lobes.

Another interesting observation was the overlapping of glandular branches, as we presented in our schematic drawing (Figure 4). Breast ducts in human females also overlap in three dimensions in superficial and deep planes, and may cross all breast quadrants [26]. We found an overlap of at least two compartments of different glands. Therefore, mastitis can simultaneously affect different compartments of one gland as well as two or more different glands [27]. Haematogenous and lymphatic spread can disseminate the inflammatory process from a primary place of origin to other nearby structures. Bacteria can also extravasate a blood vessel through the endothelial barrier and spread to healthy tissue structures, establishing new infection sites [28]. This could indicate that the vicinity and intertwining of healthy and pathogen-affected glandular lobes among different glands could result in infection dissemination.

One of this study’s limitations was its small sample size. We cannot say with certainty that individual variations, age, breed, the number of previous litters and lactations or other comorbidities did not play a role in the outcome of this casting process. Another limitation was incomplete information regarding oestrus and lactation phases. Using histological cross-sectioning, Orfanou et al. (2010) showed that canine mammary glands undergo significant changes during the lactation phase [22]. Further studies which consider these variables need to be undertaken. Nevertheless, this study’s results have significant potential for further clinical and scientific application in veterinary medicine.

## 5. Conclusions

The canine mammary gland consists of multiple compartments. Glandular and ductal branches can interlace with branches that drain into other milk ducts, but do not directly interact with them. Orifices on the teat apex usually drain one glandular compartment; however, in this study we observed that two orifices may drain a single gland compartment. Ascendant teat ducts can be solitary or split into two or three narrower ducts and can drain glands or end blind. These ducts can have the same diameter along their entire length, or appear as an imperceptibly or slightly wider canal in their proximal part before they start to branch. Mammary glands can develop differently, but whether individual variations occur regardless of the oestrus or lactation phase cannot be addressed without more detailed investigation.

This study sought to fill gaps in our knowledge regarding the anatomy, morphology and physiology of the canine mammary gland ductal system. Lack of genuine photos of the fine anatomical inner and hidden structures, anatomical diagrams and descriptions of the glands’ gross anatomy encouraged implementation of the intraductal approach. This method excellently replicated the forms of branching features of cannulated ducts. A better understanding of the anatomical structures related to the canine mammary gland could clarify the dissemination process of bacterial mastitis, and might aid the surgical and medical management of neoplastic and non-neoplastic mammary disorders.

## Data Availability

Data sharing is not applicable to this article.
